# Dendritic Cell-Derived Exosomes in Cancer Immunotherapy

**DOI:** 10.3390/pharmaceutics15082070

**Published:** 2023-08-01

**Authors:** Shumin Luo, Jing Chen, Fang Xu, Huan Chen, Yiru Li, Weihua Li

**Affiliations:** 1 Beijing Institute of Hepatology, Beijing Youan Hospital, Capital Medical University, Beijing 100069, China; luo602123@163.com (S.L.); 18510688677@163.com (J.C.); xufangmed@163.com (F.X.); 18500399656@163.com (Y.L.); 2 Integrated Chinese and Western Medicine Center, Beijing Youan Hospital, Capital Medical University, Beijing 100069, China; 18801126817@163.com

**Keywords:** dendritic cells, exosomes, cancer, immunotherapy

## Abstract

Exosomes are nanoscale vesicles released by diverse types of cells for complex intercellular communication. Numerous studies have shown that exosomes can regulate the body’s immune response to tumor cells and interfere with the tumor microenvironment (TME). In clinical trials on dendritic cell (DC)-based antitumor vaccines, no satisfactory results have been achieved. However, recent studies suggested that DC-derived exosomes (DEXs) may be superior to DC-based antitumor vaccines in avoiding tumor cell-mediated immunosuppression. DEXs contain multiple DC-derived surface markers that capture tumor-associated antigens (TAAs) and promote immune cell-dependent tumor rejection. These findings indicate the necessity of the further development and improvement of DEX-based cell-free vaccines to complement chemotherapy, radiotherapy, and other immunotherapies. In this review, we highlighted the recent progress of DEXs in cancer immunotherapy, particularly by concentrating on landmark studies and the biological characterization of DEXs, and we summarized their important role in the tumor immune microenvironment (TIME) and clinical application in targeted cancer immunotherapy. This review could enhance comprehension of advances in cancer immunotherapy and contribute to the elucidation of how DEXs regulate the TIME, thereby providing a reference for utilizing DEX-based vaccines in clinical practice.

## 1. Introduction

The field of immunotherapy for cancer has noticeably attracted oncologists’ attention since it was discovered in the late 19th-century [1,2]. Compared with traditional approaches that target both cancer cells and healthy cells, immunotherapy for cancer possesses the advantages of targeting tumor cells specifically, causing fewer side effects, stimulating immunity, and killing both primary and metastatic tumor cells [3,4]. Dendritic cells (DCs) play an important role in immunotherapy [5,6]. They present antigens to innate and adaptive immune systems, and as a result, they are considered key targets for the development of cancer vaccines [6]. However, in clinical trials, DC-based tumor vaccines have not yet met expectations [7]. It has been demonstrated that DC-derived exosomes (DEXs) play an important role in inhibiting tumor growth and development and inducing immunotherapy [8,9]. Mature DEXs express major histocompatibility complex class I and II (MHC-I and MHC-II) as well as co-stimulators that activate antigen-specific T cells against tumors [10]. Single subcutaneous injection of DEXs can suppress tumor growth and even eradicate established tumors in mice [11]. Due to their greater resistance to tumor immunosuppression, DEXs are currently considered a viable alternative to DC-based vaccines in clinical and preclinical trials for melanoma, non-small-cell lung cancer, and hepatocellular carcinoma, as well as animal studies [12,13,14,15,16]. Additionally, some patients continued to receive DEXs for four months, during which time the condition was stabilized [12]. Moreover, as biological agents, DEXs can be strictly regulated in the manufacturing process (e.g., their composition and MHC-I and MHC-II content can be easily defined), and they do not have the risk of in vivo replication that is associated with cell-based therapies [17]. In contrast to other anticancer vaccines, treatment with cell-free DEXs can resist the immune modulation that occurs in tumors [18]. Moreover, DEX-based antitumor vaccines have also proved to be more effective against tumors in preclinical animal models [19,20]. In conclusion, DEXs have great potential in the field of immunotherapy for cancer, and their significance for disease treatment and vaccine development should be explained and clarified further. This article introduces the latest research progress of DEXs in tumor immunotherapy, emphatically introduces the biological characteristics and production mechanism of DEX, and outlines the important role of DEX in the tumor immune microenvironment (TIME), such as the B and T cell activation mechanism and clinical application of DEX in non-small-cell lung cancer, melanoma, liver cancer, and other tumor immunotherapy.

## 2. Biogenesis and Biological Functions of Exosomes

Exosomes are micro-vesicles with a diameter of 30–150 nm and a density of 1.13–1.19 g/mL, secreted by cells with a phospholipid bilayer structure [21]. In 1983, exosomes were first assumed to be vesicles released by sheep reticulocytes that eliminated unnecessary or undesired cellular components, such as transferrin receptor [22,23,24,25,26]. Various cells can secrete exosomes into body fluids, including blood, urine, saliva, tears, and cerebrospinal fluid, releasing exosomes into the extracellular environment via specific mechanisms [25]. In 1996, Raposo et al., revealed that B lymphocytes may produce antigen-presenting vesicles, which then excite CD4^+^ effector T cells, resulting in a superior antitumor immunity [26]. As scientists learned more about exosomes, they realized how important exosomes are for intercellular communication and regulation of the internal environment of cells. The safety, satisfactory biodegradability, and promising biocompatibility of biogenic nanocarriers make them valuable targets for cancer therapy [19]. Exosomes provide an ideal microenvironment for the effective action of immunomodulators. They can also deliver antigens; influence tumor cell proliferation, invasion, and migration; and stimulate tumor-specific immune responses [27,28]. To date, exosomes have been used as sensitive biomarkers for the diagnosis of cancer [29]. Hence, the use of exosomes as therapeutic targets may be a better approach to initiating immune responses against tumors [30,31].

Secretion of exosomes is not a random process, and it necessitates the close coordination of several components. Similar to apoptosis, the production of exosomes entails the flipping of phosphatidylserine from the inner to the outer plasma membrane leaflet [32,33]. Production of exosomes is initiated by endocytosis at the cell membrane’s surface, followed by inward budding to generate early-sorting endosomes [34]. The endosomal membrane folds and invaginates as it matures towards late-sorting endosomes, encasing particular proteins, nucleic acids, and other components, and finally produces multivesicular bodies (MVBs), containing intraluminal vesicles (ILVs) [35]. Some MVBs are then fused with lysosomes and disintegrated, whereas another proportion of MVBs with CD63 and lysosome-associated membrane proteins on the membrane surface can facilitate their fusion with the cell membrane [33]. The process of maturation of MVBs to the release of ILVs into the extracellular environment is primarily governed by two mechanisms: the endosomal sorting complex required for transport (ESCRT)-dependent route and the ESCRT-independent pathway [36]. ESCRT proteins are made up of ESCRT-0-III and 30 other proteins, including VPS4, VTA1, apoptosis-linked gene 2-interacting protein X (ALIX), etc. They are involved in endosomal membrane invagination, MVB production, and MVB fusion with cell membrane, finally releasing exosomes via cytokinesis. Rab proteins, such as Rab 27a, Rab27b, Rab35, and Rab11, are involved in ILV creation and sorting of proteins and biomolecules [37]. Hepatocyte growth factor-regulated tyrosine kinase substrate (HRS) is a component of ESCRT-0 that identifies ubiquitinated proteins and interacts with STAM, another component of ESCRT-0 [38]. HRS plays a key role in exosome formation, and it has been shown that HRS is essential for DC-secreted exosomes, which is noticeably reduced in HRS-depleted DCs, and antigen-presenting function is hindered [39,40]. The endosomal membrane can wrap certain molecules for budding when ESCRT-I and ESCRT-II work together. ESCRT-III is hypothesized to play a role in the budding process, and it is essential for the conversion of ILVs to MVBs [41,42].

**Figure 1:**
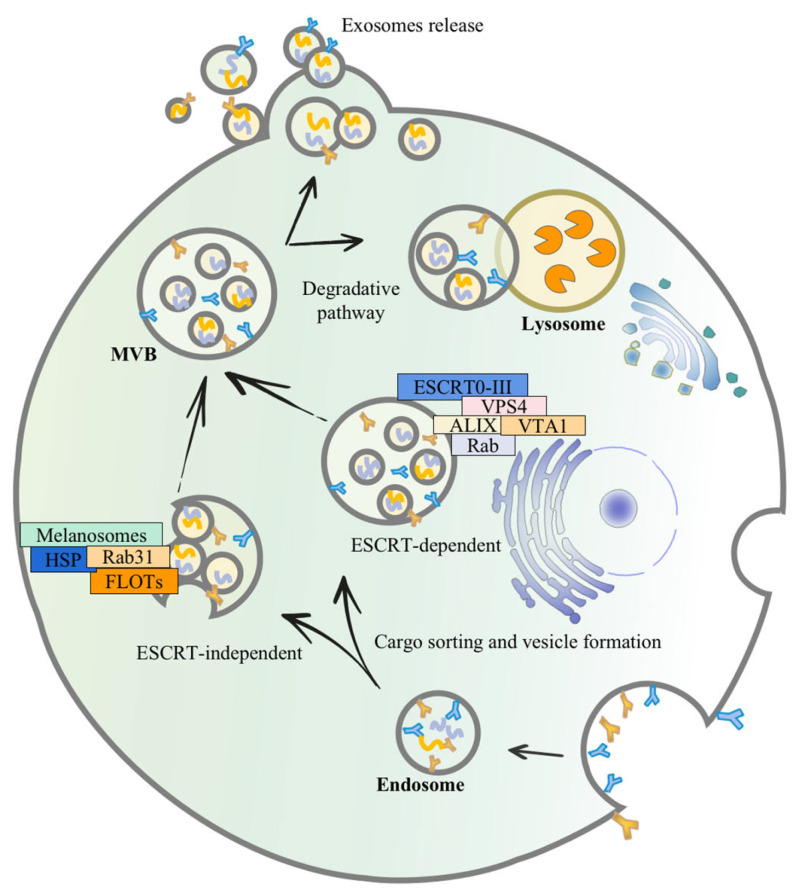
The mechanism of exosome secretion. Exosomes budding inward through restricted endosomal membranes form early endosomes, which fold, invaginate, and encapsulate specific proteins, nucleic acids, and other materials, eventually forming multivesicular bodies (MVBs) containing intraluminal vesicles (ILVs). A part of the MVBs can be degraded by binding to lysosomes; it fuses with the plasma membrane, releasing these ILVs as free exosomes into the extracellular environment. One of the mechanisms of exosome biogenesis is an ESCRT-dependent pathway which mainly involves 30 proteins, including ESCRT0-III, VPS4, VTA1, and ALIX. The other is an ESCRT-independent pathway, mainly involving melanosomes, raft-based microdomains, lipids, tetramembrane, HSP, RAB31, and FLOTs.

MVBs may also be formed without the use of ESCRT, and the lateral segregation of cargo inside the endosomal membrane appears to be dependent on raft-based microdomains, lipids, the tetramembrane protein family, and the heat-shock protein family (HSP) [35]. Tetraspanin-enriched microdomains (TEMs) and tetraspanin work together to sort target receptors and intracellular components into exosomes [43]. Regarding the ESCRT-independent pathway, knockdown of components of ESCRT and related proteins did not affect the generation of Rab31-driven epidermal growth factor receptor (EGFR) exosomes [44]. As a result, Kang et al., discovered a Rab31 that controlled the ESCRT-independent route. Rab31 can accelerate the creation of ILVs and inhibit the degradation of MVBs during exosome biogenesis. After interacting with flotillin (FLOT) proteins in the microstructural region of lipid rafts, activated Rab31 promotes MVB budding, while inactivated Rab7 prevents MVBs from fusing with lysosomes, enabling ILVs to be released via the fusion of MVBs with cell membranes [44]. In addition, melanosomes in pigment cells have a specialized endosome/lysosomal structure with an MVB-like structure that can localize ILVs without the ESCRT process, via an ESCRT-independent exosome production pathway. Furthermore, certain cells with ESCRT key factor mutations may still shape ILVs, owing to the spontaneous deformation of lipid structural domains in the endosomal membrane and isolation, although this exosome creation is less effective [45]. Specialized processes can be found in diverse types of cells to ensure the specific sorting of bioactive constituents into exosomes, extending our knowledge about exosome production pathways (Figure 1).

## 3. The Importance of Biological Messages Carried by DEX in Cancer Immunotherapy

### 3.1. Components of DEX

**Figure 2:**
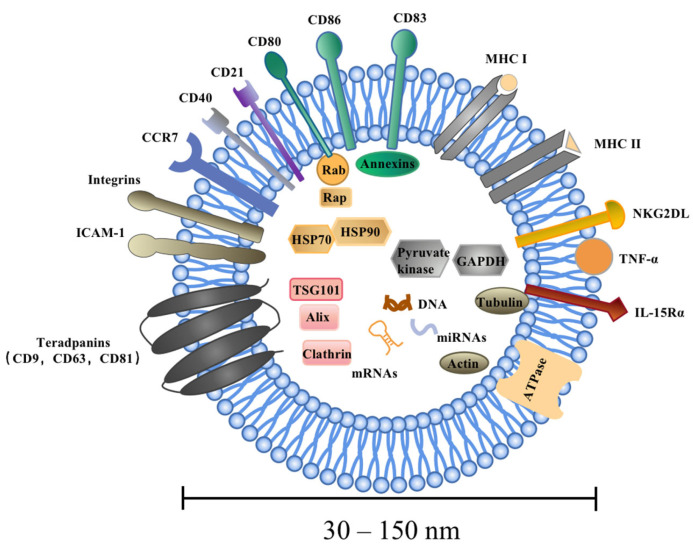
Components of DEXs. DEXs are highly heterogeneous and range in size from 30 to 150 nm. DEXs are composed of a membrane lipid-bilayer and are packed with cytosol containing soluble proteins, small molecules, and genetic information. There are three main components of a DEX, namely proteins, lipids, and nucleic acids, mainly including the tetraspanin (CD9, CD63, and CD81), Alix, Tsg101 and heat-shock proteins (HSP70, HSP90), as well as integrins, cytoskeletal proteins, signaling proteins, DNAs, mRNAs, miRNAs, and lncRNAs. DEXs contain abundant membrane surface parental DC molecules, such as MHC-I, MHC-II, CD80, CD86, NKG2DL, IL-15Rα, CD40, ICAM-1, CD21 CD11c, CD83, CCR7, etc., confirming the potent antitumor immune effects of DEXs.

Similar to DCs, the molecular composition of DEXs includes surface expression of functional MHC-peptide complexes, costimulatory molecules, and other components that interact with immune cells [46,47]. DEXs are composed of a membrane lipid-bilayer and are packed with cytosol, containing soluble proteins, small molecules, and genetic information [8]. The vesicle membrane has the same geometry as the plasma membrane of DCs, allowing the external exposure of extracellular domains of transmembrane proteins to interact with their respective partners on target cells [48]. To date, it has been found that DEXs contain abundant MHCI and MCHII molecules, such as MHC-I, MHC-II, CD80, CD86, NKG2D, IL-15Rα, CD40, ICAM-1, CD21, CD11c, CD83, and CCR7, confirming the potent antitumor immune effects of DEXs [49,50]. Recently, DEXs have been found to deliver tumor necrosis factor-α (TNF-α) [51,52]. These findings provide opportunities for further development of DEX-based vaccines. However, the exact composition of EVs reflects the site of origin of DEXs with endosomal membrane rafts enriched in endosome-derived EVs as exosomes [53]. Three main components of EVs, including proteins, lipids, and nucleic acids, are discussed in the present study. Exosomes contain specific cellular proteins, some of which are determined by the cell type that may secrete them. Firstly, DEX are abundant in proteins, mainly including immunostimulation-related proteins, adhesion and targeting-related proteins, cytoskeletal proteins, membrane transport and fusion proteins, anti-apoptosis-related proteins, etc. [10,20,37]. Secondly, lipids wrap around soluble cellular contents and form the outer membrane of exosomes. Exosomes of different cellular origins are enriched in glycosphingolipids, sphingomyelin, phosphatidylserine (PS), cholesterol, and ceramide, constituting the specific lipid composition of exosomes [46]. Thirdly, mRNAs, microRNAs (miRNAs), short non-coding RNAs, and DNAs have been identified in exosomes and may regulate genes in recipient cells, such as miRNAs and their immature form (pre-miRNAs) [54,55]. The specific components of DEX are listed in Table 1 and Figure 2.

### 3.2. Functions of DEXs

DEX-based therapy may be a promising approach to initiate antitumor immune responses [13]. DEXs possess unique compositional characteristics that allow for the delivery of cargo to target cells. DEXs can be delivered to lymph nodes after they have been released to trigger certain cellular immune responses [10]. T cells can be triggered to differentiate into Th1, Th2, Treg, or Th17 phenotypes based on the signals released by DEX surface-active ingredients. T cell activation induced by DEXs is important in cancer immunotherapy, and DEXs also express natural killer (NK) receptors, causing activation of NK cells [25,59,60].

Because of their different compositions, immature and mature DEXs have distinct biological activities [61]. Immature DEXs (ImDEXs) have substantially reduced amounts of molecules, such as MHCI/II, CD86, and ICAM-1, which may be involved in direct T cell activation or DC targeting [62]. As a result, imDEXs have shown a poor immunogenicity in tumor-bearing mice and patients. ImDEXs have been utilized for cell-free vaccination in patients with advanced melanoma and non-small-cell lung cancer (NSCLC) in early clinical tests [63], which is likely one of the factors for the limited specific T cell responses. When DCs are activated by maturation signals, including the production of damage-associated molecular paradigms (DAMPs) and pathogen-associated molecular paradigms (PAMPs), they achieve a mature state. They then participate in the immune system’s response [50]. Exosomes contain antigenic peptides released by mature DCs that may promote more effective and specific immune activators to detect and kill tumor cells and pathogens [7,64]. Mature DCs release 50–100 times more exosomes than immature DCs, and exosomes secreted by mature DCs play a key role in antigen presentation to cytotoxic T lymphocytes (CTL) [65,66].

### 3.3. Immunotherapeutic Benefits of DEXs 

Compared with DC-based vaccines, DEXs provide additional benefits [13]. Currently, the process of generating DEX vaccines from cells derived from PBMC can be performed in a GMP (Good Manufacturing Practices)-compliant laboratory in a cell therapy unit [13]. High-purity DEXs can be obtained via differential centrifugation, and the structure and size of DEXs can be visualized using a NanoSight instrument and transmission electron microscopy (TEM) [67]. Imaging flow cytometry can also be used to quantify cytoplasmic proteins from parent cells and exosomes markers such as CD9, CD63, and CD81 [68]. Studies have shown that exosomes produced in GMP plants remain active after three or six months of freezing at −80 °C [69]. Moreover, the homogeneity of exosomes from different batches has been well characterized, demonstrating the feasibility of large-scale production of exosomes [68]. Significant antitumor efficacy has been achieved in clinical trials with DEX vaccines based on pDC cell lines, and the establishment of DC cell lines rather than auto-MoDC remains a promising approach for future DEX vaccine preparation and application [70,71]. As a result, the molecular composition of DEXs is more restricted than that of DCs. DEXs provide easy transportation and long-term storage while maintaining phenotype and function [72]. Moreover, DEX particles are smaller than DCs and can more efficiently reach the relevant places in secondary lymphoid organs with target cell selectivity [73]. They can also penetrate a variety of biological barriers, including the blood–brain barrier and the blood–tumor barrier [8]. Based on this feature, it may be possible to use DEXs for the treatment of various brain diseases such as brain tumors and cerebrovascular diseases in the future [74]. DEX-tracking experiments have confirmed that DEXs are mainly distributed to the tumor site as well as the liver in tumor-bearing mice [16]. Moreover, DEX-based vaccines are remarkably safer than DC-based vaccines. Tumor-associated monocyte-derived DCs (MoDCs) possess a suppressive TIP-DC phenotype, preventing T cells from being potently activated by high expression levels of inducible nitric oxide synthase (iNOS) and TNF-α. Immune regulatory cells and immunosuppressive molecules can also reduce the functions of DCs, which is common in cancer patients [75]. However, DEXs, as inert vesicles, are either not or less sensitive to immunosuppression [75,76]. DEXs may be more effective in activating T and NK cells than DCs [50,77,78]. This is because the MHCs on the surface of DEXs are preferentially enhanced, and the surface of DEXs has been demonstrated to be enriched in NK cell-activating ligands [57]. Exosomes are a subcellular structure, and there are no ethical restrictions associated with their clinical application. DEXs are without nuclei and cannot differentiate, preventing tumor transformation and aberrant differentiation [62]. In preclinical trials, DEX-containing tumor antigens were found to have a higher anticancer effectiveness than DC-based vaccines in eliminating existing murine tumors [60,79].

### 3.4. The Role of DEXs in Cancer Immunotherapy

The development and progression of cancer depend on its complex and heterogeneous microenvironment, which is composed of both cellular and cell-free components [80]. An essential role of DEXs in cancer progression is to regulate tumor immune responses, participating in multiple stages of tumor development and progression [30]. DEXs can be involved in tumor immunotherapy by regulating energy metabolism, mediating the inflammatory microenvironment and intercellular communication, providing stimulatory signals, etc. [5,81]. DEXs regulate the immune response in the body by presenting MHC and antigen complexes directly to T cells or indirectly to surrounding antigen-presenting cells (APCs) [82,83,84]. Additionally, DEXs can directly induce proliferation and activation of NK cells via the expression levels of surface proteins, including natural killer group 2 member D ligands (NKG2DL) and interleukin (IL)-15Rα (IL-15Rα) [85].

### 3.5. DEXs Trigger T Cell Immune Responses

DEXs can induce T cell immune responses via direct and indirect pathways [86]. On the one hand, DEXs can directly stimulate T cells in vitro, while MHC-I and MHC-II molecules on the surface of DEXs make it possible to directly stimulate CD8^+^ and CD4^+^ T cells, respectively [57,86]. DEXs can improve the response efficiency of activated T cells by immobilizing DEXs, increasing their in vitro concentration, or loading them with antigenic peptides [87]. On the other hand, DEXs do not directly stimulate T cells while transferring tumor antigen peptides and MHCs to other APCs. Indirect antigen presentation has a stronger stimulatory effect on T cell immune responses [82]. There are two mechanisms for the indirect stimulation of T cells. The first mechanism is the transfer of MHC molecules and tumor antigen peptides from exosomes endocytosed by DCs. Subsequently, these MHC/peptide complexes are transported to the DC surface for presentation to T cells. Previous studies have suggested that immature DCs are more likely to internalize DEXs, while mature DCs reserve DEXs on the surface [72].

The second mechanism is the direct transfer of DEX-containing MHC/peptide complexes to the surface of bystander APCs, initiating antitumor immune responses in a process called cross-presentation [82,84]. After internalizing DEXs, APCs reprocess the MHC/peptide complexes via the endosomal pathway, and finally transport the complex to the DC surface for presentation to T cells. Additionally, DEXs are capable of triggering T cell responses via tumor cells. DEXs with MHC-peptide complexes can transfer to the surface of tumor cells and reverse their immunogenicity, thereby allowing tumor cells to be directly targeted by host T cells [77,80]. Importantly, the efficiency of indirect activation of T cells seems to be highly dependent on the activation state of DCs. Exosomes released from DCs stimulated with lipopolysaccharide (LPS) or interferon-gamma (IFN-γ) increased the surface expression of costimulatory factors and had a greater triggering effect on CD8^+^ T cell immunity [64,88].

### 3.6. DEX-Induced Activation of NK Cells

Studies have demonstrated that DEXs have ligands for members of NKG2D-L and IL-15Rα, both of which activate NK cells and stimulate innate immune responses [59,78]. This finding highlights the possibility that DEXs activate both adaptive and innate responses, and NK cells can activate tumor immune responses in vitro through disseminated metastases [18]. Furthermore, DEXs can activate NK cells and trigger caspase-mediated tumor cell apoptosis in vitro by expressing tumor necrosis factor-related apoptosis ligand (TRAIL), tumor necrosis factor (TNF), and FasL [52]. In a mouse model of advanced melanoma, DEXs could induce IL-15Rα and NKG2D-dependent proliferation of NK cells and promote IFN-γ release, leading to a metastatic effect of NK cells in the local TME [12,14]. Similar to DCs, DEXs express Toll-like receptor 4 (TLR4) and Toll-like receptor 1/2 (TLR1/2) ligands on their surface, upregulate the expression level of TNF, and interact with TNF receptors on NK cells that may affect the production of IFN-γ by NK cells [89,90].

### 3.7. DEXs Interact with B Cells

**Figure 3:**
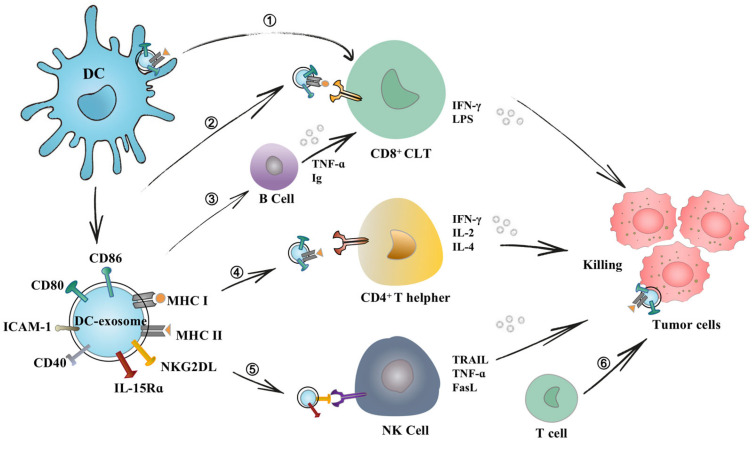
Interaction of dendritic cell exosomes with immune cells. DEXs mediate adaptive and innate immune responses via direct and indirect pathways. ➀ DEXs adhere to the surface of DC cells, presenting antigens and activating T cells. ➁ Immune-related molecules on the surface of DEXs also enable them to directly stimulate CD8^+^ T cells and secrete IFN-r, which plays a major antitumor effect. ➂ DEXs were found to present B cell co-stimulatory molecules (CD54), contributing to B cell recognition of epitopes. ➃ DEXs directly present pMHC complexes to CD4^+^ T cells to assist T cells in killing tumor cells. ➄ DEXs have also been shown to possess NKG2DL and IL-15Rα complexes, which can enhance the function of NK cells and thus activate antitumor immune responses. ➅ They can transfer MHC and antigenic peptide complexes to the surface of tumor cells, allowing host T cells to target tumor cells and incurring immunogenic cell death.

A growing body of evidence demonstrates that DEXs can induce B cell immunity in vitro and in vivo. Segura et al., showed that DEXs can indirectly activate T cells by delivering antigen-MHC combinations and ICAM-1 molecules to less efficient APCs, such as B cells [66,91]. Activated B cells synthesize and release exosomes that can efficiently stimulate both CD4^+^ T cells and CD8^+^ T cells to respond efficiently to cancer cells and elicit tumor immune responses [92]. It is noteworthy that B lymphocytes perform several functions, including the presentation of Ags to T cells, differentiation of T lymphocytes into follicular T helper lymphocytes, and Ag transport [93]. B cells are essential for the optimal triggering of CD8^+^ T cells via exosomes [92]. In an in vitro study, Quah et al., showed that exosomes secreted by mycoplasma-contaminated DCs are potent mitogens for B cells, leading to polyclonal activation of B cell subsets and promoting Ig secretion [94]. In a mouse model, Qazi et al., showed that OVA-DEXs contributes to the accumulation of complex factors, such as C3 or C4, which confers a surface of DEXs on B cells using CD21 and ICAM-1 molecules (co-stimulatory molecules for B cells) [95]. In exploring anti-graft immune responses, DEXs were shown to initiate the production of IgG2a and B antibodies (type I antibodies) in vivo and prolong the survival of alloantigens [96]. The above-mentioned findings highlighted the importance of DEXs for initiating B cell immune responses. The main interaction of dendritic cell exosomes with immune cells is shown in Figure 3.

### 3.8. Role of DEXs-Based Immunotherapy in Different Tumors

After a DEX-based antitumor vaccine was tested in animal models, phase I and phase II clinical trials were completed in patients with advanced cancer. In addition, additional animal experiments on different tumors related to DEXs for immunotherapy were carried out, aiming to produce a new generation of DEX-based antitumor vaccines with higher biological activity.

### 3.9. DEXs and Melanoma

Clinical trials on DEXs are currently in progress. Exosomes isolated from DCs were derived from immature monocytes of melanoma patients and were stimulated by melanoma-associated antigenic peptides (MAGE). Improved immunity to melanoma has been found after patients were inoculated with self-derived DEXs. The study showed that in 15 patients with metastatic melanoma, DEXs had no secondary toxicity at the maximum tolerated dose, demonstrating that auto-DEXs with MAGE would be tolerated well in melanoma patients, and this tolerance was strongly associated with the enhanced activity of NK cells [14]. The results showed that the DEX-based antitumor vaccine induced NK cells to proliferate in an IL-15Rα-dependent manner [59,78]. Meanwhile, cytokines produced by NK cells are directly related to the expression level of exosomal BAG-6 [18,78]. NKG2DL, TNF, FasL, and TRAIL are expressed on the surface of DEXs, activate NK cells, and stimulate the secretion of IFN-γ [52,90].

It has been demonstrated that tumor vaccines for patients require the enhanced biological activity of DEX-stimulated T cells in vivo. In a mouse model, DEXs stimulated by antigens from human B16F10 melanoma cell lysates could consistently induce activation of melanoma-specific CD8^+^ T cells and recruit NK cells to tumor sites. This significantly inhibits tumor growth and prolongs the survival of tumor-bearing mice [89].

Recently, Charles et al. [71] reported the results of a phase I clinical trial in patients with metastatic melanoma, in which pDCs were derived from human pDC cell lines rather than from cancer patients. Therefore, future studies should concentrate on whether these cell lines can replace DCs isolated from autologous cells and utilize them for the large-scale production of DEXs. This indicates that tumor vaccines can be obtained in a low-cost, unlimited manner with an easier quality control [97]. This research may enrich cancer immunotherapy options without cellular vaccines.

### 3.10. DEXs and Non-Small-Cell Lung Carcinoma (NSCLC)

MAGE-specific T cell responses, along with the increased natural killer cell lysis activity, were observed in a phase I trial in patients with NSCLC [14,16], demonstrating that DEX-based immunotherapy is safe and well-tolerated. Besse et al., improved the DEX-based antitumor vaccine, which was tested in a phase II clinical trial on patients with advanced NSCLC, and it was found that DEXs improve antitumor immune ability in patients with advanced NSCLC [15].

An essential innovation in the phase II DEX immunotherapy trial was the use of DEXs from TLR4 ligand (TLR4L) or IFN-γ-stimulated DEXs (IFNγ DCexos). Compared with DEXs from autologous immature monocytes, such DEXs expressed more co-stimulatory molecules and induced a higher immunostimulatory response [64]. The results of the phase II trial suggested that IFN-γ-DEXs can be a well-tolerated immunotherapy. The research not only confirmed that the production of IFN-γ-DEXs is feasible, but also revealed that immunostimulatory effects may be dependent on NK cell surface receptor NKp30 signaling, which enhances activation of NK cells [88]. However, no significant antigen-specific T cell response was found in this trial. Possible explanations given by the investigators include the lack of collectible T cells in circulation, possibly due to T cell migration to tumor sites, and other reasons, such as the heterogeneity of the cohort of patients with advanced disease, or regulatory mechanisms (e.g., Treg activity), hindering the widespread utilization of the immunotherapy [10]. DEX-based immunotherapy can enhance antigen-specific T cell responses.

### 3.11. DEXs and Hepatocellular Carcinoma (HCC)

DEXs have recently shown higher feasibility and efficacy in HCC patients in preclinical trials. Alpha-fetoprotein (AFP) is currently one of the most commonly measured clinical biomarkers for HCC [98]. DEX-expressing AFP can promote antigen-specific immune response, suppress tumor growth, and alter the TME by increasing the expression levels of IFN-γ, interleukin-2 (IL-2), and CD8^+^ T lymphocyte, as well as decreasing the levels of regulatory T cells (Treg), interleukin-10 (IL-10), and transforming growth factor-β (TGF-β) [99]. The antitumor effect mediated by DEXs_AFP_ was positively correlated with the improvement of the TIME in HCC mice, and T cells contributed to the antitumor function of DEXs_AFP_. Based on these results, DEXs_AFP_ have a potential clinical value as a novel class of vaccines for the immunotherapy of hepatocellular carcinoma, and the dose and delivery method of DEXs should be optimized in clinical practice. As research progresses, DEXs may be soon used in the treatment of HCC [41,99,100,101,102].

Shi et al., showed that in a mouse model of HCC, the use of sorafenib and stimulation of DCs with exosomes from tumor cells reduced the number of regulatory T cells and increased the count of CD8^+^ T cells [103]. Liugang et al., designed artificial cell membrane nanoparticles derived from DCs with good stability and homing effect to rapidly present new antigens to CD8^+^ T cells and stimulate a strong CTL response, thereby activating a strong antitumor immune response [104]. Meanwhile, when PD-1 antibodies were used to block the PD-1/PD-L1 pathway, this combined therapeutic strategy had a favorable antitumor effect [103,104,105]. An experiment also showed that the combination of microwave ablation and DEXs enhanced the antitumor effect in tumor-bearing mice and significantly suppressed the growth of HCC cells by improving the TIME compared with microwave ablation alone [106]. The next generation of DEX-based antitumor vaccines is intended to be used in combination with multiple cancer therapies for greater antitumor efficacy. At present, another increasing approach of DEX tumor vaccines is to use altered antigen ligands or neoantigens to co-incubate with DEXs to bind specific antigens that deliver DEXs specifically to tumors. This is a promising sign of our ongoing work, utilizing DEXs to bind with short peptides of liver cancer-specific AFP and GPC3 that can more specifically activate T lymphocytes for prevention or targeted therapy of liver cancer.

## 4. Conclusions

To date, DEXs have not achieved the expected therapeutic effects in clinical practice. The main limitations of this immunotherapy are the lack of pre-selection criteria for recruiting patients with advanced cancer and the heterogeneity of several patients who have already received immunotherapy. Secondly, patients’ partial or systemic immunoregulatory mechanisms also limit this immunotherapy. The biological activity of DEXs in mediating the tumor immune response requires further improvement. Thirdly, standardized isolation and highly quantitative exosomes are required due to the poor purity of DEXs and the limited amount of DEXs secreted by the cells [36]. The development of stable pDC cell line vaccines could significantly boost the antitumor effect in clinical trials [62,71]. Quality control criteria for DEX-based antitumor vaccines include the expression levels of tetraspanins (CD9, CD63, CD81, and CD82) and HLA-DR, and other exosomal markers (Tsg101 and HSP70) [107].

Despite several challenges in DEXs-based immunotherapy, this approach is still a promising cancer treatment. The biological activity of DEXs can be modulated via various methods, such as modification of exosome-derived cells by genetic engineering and promotion of DEXs production by light sensing based on phototherapy. A previous study showed that the amount of DEXs secreted from vesicles in DCs can be remarkably increased more than 13-fold with phototherapy-based LED lights under more superior light wavelength, intensity, and exposure time conditions [108]. At present, a new generation of DEX-engineered vaccines is under development, which is expected to improve the expression levels of surface costimulatory molecules of DEXs and reduce the expression levels of immune-regulatory molecules, such as programmed death-ligand 1 (PD-L1) and Tregs, to reduce immunosuppression [104,105]. Moreover, the selection of tumor antigenic peptides (TAA) could be improved. The selection of an appropriate antigenic peptide causes DCs to secrete highly specific exosomes, which may contribute to the development of DEX-based antitumor vaccines with more specific immune responses.

DEX-based immunotherapy is a valuable strategy that can be used alone or in combination with other cancer treatments to replace or augment existing immunotherapies. More animal tests with DEX cell-free vaccines targeting various malignancies should be conducted, demonstrating the potency of DEXs. Prospects for this immunotherapy aim at developing high-immunogenicity DEXs with genetic modifications to achieve a larger scale of cell-free immunotherapy. Furthermore, the use of immune adjuvants and their combination with other cancer therapeutic modalities may enable DEXs to stimulate T cells more effectively in the future [71,103,106,109]. In conclusion, DEX-based antitumor vaccines are still in the preliminary stage of exploration. Their complex immune mechanisms have not yet been fully explored, and further research is required to improve the understanding and application of DEXs.

## Figures and Tables

**Table 1:** Components of DEXs and their markers and functions.

Components	Markers	Functions
Immune-stimulation-associated proteins	HLA-ABC, HLA-DP/DQ/DR, CD80, CD86, CD40, NKG2DL, IL-15Rα	Activation of CD8^+^ T and CD4^+^ T cells [56,57].
Adhesion and targeting associated proteins	Integrin α and β chains (αMβ2), ICAM-1, MFG-E8, lactadherin, CD1a, b, c, and d proteins	DEX are targeted to the destination and recipient cells, cross-presentation of lipid antigens [20].
Cytoskeletal proteins	Microtubulin, actin, actin-binding protein	Maintaining the normal morphology of the DEX, withstanding certain external forces, and maintaining the normal functioning of the various functions of the DEX [19].
Membrane transport and fusion proteins	Annexins, RAB proteins and HSP, CD9, CD37, CD63, CD81, CD82, Flotilin 1 and 2	Assists MHC molecules to load antigens that enhance DEX immunogenicity by triggering an immune response, clearance of misfolded proteins, stabilizing proteosomes, and protecting cells from stress [58].
Anti-apoptosis associated proteins	Thioredoxin peroxidase II, Alix, 14-3-3, and galectin-3	Anti-apoptotic.
Exosome secretion-related proteins	Tumor susceptibility gene 101 (TSG101)	Determining the sorting of ubiquitinated cargos into ILVs during exosome generation.
Lipids	Phosphatidylserine, cholesterol, ceramide, sphingomyelin and glycerophospholipids, lysobisphosphatidic acid (LBPA)	Major components of the exosome membrane, conferring stability onto exosomes, the mechanism of exosome formation, release and function [58].
Nucleic acids	mRNA, miRNA and other non-coding RNAs, DNA	Intercellular communication and induction of certain posttranslational modifications in the recipient cells, diagnostic markers [39].

## Data Availability

Data sharing is not applicable. No new data were created or analyzed in this study.

## Abbreviations

TME	Tumor microenvironment
DEXs	DC-derived exosomes
TAAs	Tumor-associated antigens
TIME	Tumor immune microenvironment
MVBs	Multivesicular bodies
ILVs	Intraluminal vesicles
ESCRT	Endosomal Sorting Complex Required for Transport
VPS4	Vacular sorting protein 4
ALIX	Apoptosis-linked gene 2-interacting protein X
HRS	Hepatocyte growth factor-regulated tyrosine kinase substrate
STAM	Signal transducing adaptor molecule
TEMs	Tetraspanin-enriched microdomains
HSP	Heat-shock protein family
FLOT	Flotillin
EGFR	Epidermal growth factor receptor
NKG2D	Natural killer group 2 member D
ICAM-1	Intercellular cell adhesion molecule-1
TNF-α	Tumor necrosis factor-α
PS	Phosphatidylserine
LBPA	Lysobisphosphatidic acid
NSCLC	Non-small-cell lung cancer
DAMPs	Damage-associated molecular paradigms
PAMPs	Pathogen-associated molecular paradigms
CTL	Cytotoxic T lymphocytes
MoDCs	Monocyte-derived DCs
iNOS	Inducible nitric oxide synthase
APCs	Antigen-presenting cells
NKG2DL	Natural killer group 2 member D ligands
LPS	Lipopolysaccharide
IFN-γ	Interferon-gamma
TRAIL	Tumor necrosis factor-related apoptosis ligand
TLR4	Toll-like receptor 4
TLR1/2	Toll-like receptor 1/2
OVA	Ovalbumin
BAG-6	BCL2-associated Athanogene 6
MAGE	Melanoma-associated antigenic peptides
HCC	Hepatocellular carcinoma
Treg	Regulatory T cell
IL-10	Interleukin-10
TGF-β	Transforming growth factor-β
Tsg101	Tumor susceptibility gene 101 protein
PD-L1	Programmed death-ligand 1

## References

[1] Yang P., Peng Y., Feng Y., Xu Z., Feng P., Cao J., Chen Y., Chen X., Cao X., Yang Y. (2021). Immune Cell-Derived Extracellular Vesicles—New Strategies in Cancer Immunotherapy. Front. Immunol..

[2] Dobosz P., Dzieciątkowski T. (2019). The Intriguing History of Cancer Immunotherapy. Front. Immunol..

[3] Liu Z., Lv J., Dang Q., Liu L., Weng S., Wang L., Zhou Z., Kong Y., Li H., Han Y. (2022). Engineering neoantigen vaccines to improve cancer personalized immunotherapy. Int. J. Biol. Sci..

[4] Wang Y., Wang M., Wu H., Xu R. (2021). Advancing to the era of cancer immunotherapy. Cancer Commun..

[5] Wculek S.K., Cueto F.J., Mujal A.M., Melero I., Krummel M.F., Sancho D. (2020). Dendritic cells in cancer immunology and immunotherapy. Nat. Rev. Immunol..

[6] Bol K.F., Schreibelt G., Rabold K., Wculek S.K., Schwarze J.K., Dzionek A., Teijeira A., Kandalaft L.E., Romero P., Coukos G. (2019). The clinical application of cancer immunotherapy based on naturally circulating dendritic cells. J. Immunother. Cancer.

[7] Hernández S.S., Jakobsen M.R., Bak R.O. (2022). Plasmacytoid Dendritic Cells as a Novel Cell-Based Cancer Immunotherapy. Int. J. Mol. Sci..

[8] Kowal J., Tkach M. (2019). Dendritic cell extracellular vesicles. Int. Rev. Cell Mol. Biol..

[9] Lindenbergh M.F.S., Wubbolts R., Borg E.G.F., Van ‘t Veld E.M., Boes M., Stoorvogel W. (2020). Dendritic cells release exosomes together with phagocytosed pathogen; potential implications for the role of exosomes in antigen presentation. J. Extracell. Vesicles.

[10] Elashiry M., Elsayed R., Cutler C.W. (2021). Exogenous and Endogenous Dendritic Cell-Derived Exosomes: Lessons Learned for Immunotherapy and Disease Pathogenesis. Cells.

[11] Anderson D.A., Dutertre C.-A., Ginhoux F., Murphy K.M. (2021). Genetic models of human and mouse dendritic cell development and function. Nat. Rev. Immunol..

[12] Morse M.A., Garst J., Osada T., Khan S., Hobeika A., Clay T.M., Valente N., Shreeniwas R., Sutton M.A., Delcayre A. (2005). A phase I study of dexosome immunotherapy in patients with advanced non-small cell lung cancer. J. Transl. Med..

[13] Pitt J.M., André F., Amigorena S., Soria J.-C., Eggermont A., Kroemer G., Zitvogel L. (2016). Dendritic cell–derived exosomes for cancer therapy. J. Clin. Investig..

[14] Escudier B., Dorval T., Chaput N., André F., Caby M.-P., Novault S., Flament C., Leboulaire C., Borg C., Amigorena S. (2005). Vaccination of metastatic melanoma patients with autologous dendritic cell (DC) derived-exosomes: Results of thefirst phase I clinical trial. J. Transl. Med..

[15] Besse B., Charrier M., Lapierre V., Dansin E., Lantz O., Planchard D., Le Chevalier T., Livartoski A., Barlesi F., Laplanche A. (2016). Dendritic cell-derived exosomes as maintenance immunotherapy after first line chemotherapy in NSCLC. OncoImmunology.

[16] Zuo B., Zhang Y., Zhao K., Wu L., Qi H., Yang R., Gao X., Geng M., Wu Y., Jing R. (2022). Universal immunotherapeutic strategy for hepatocellular carcinoma with exosome vaccines that engage adaptive and innate immune responses. J. Hematol. Oncol..

[17] Zhang B., Yin Y., Lai R.C., Lim S.K. (2014). Immunotherapeutic Potential of Extracellular Vesicles. Front. Immunol..

[18] Du Z., Huang Z., Chen X., Jiang G., Peng Y., Feng W., Huang N. (2022). Modified dendritic cell-derived exosomes activate both NK cells and T cells through the NKG_2_D/NKG_2_D-L pathway to kill CML cells with or without T_3_1_5_I mutation. Exp. Hematol. Oncol..

[19] Xia J., Miao Y., Wang X., Huang X., Dai J. (2022). Recent progress of dendritic cell-derived exosomes (Dex) as an anti-cancer nanovaccine. Biomed. Pharmacother..

[20] Nikfarjam S., Rezaie J., Kashanchi F., Jafari R. (2020). Dexosomes as a cell-free vaccine for cancer immunotherapy. J. Exp. Clin. Cancer Res..

[21] Anand S., Samuel M., Kumar S., Mathivanan S. (2019). Ticket to a bubble ride: Cargo sorting into exosomes and extracellular vesicles. Biochim. Biophys. Acta Proteins Proteom..

[22] Hussen B.M., Faraj G.S.H., Rasul M.F., Hidayat H.J., Salihi A., Baniahmad A., Taheri M., Ghafouri-Frad S. (2022). Strategies to overcome the main challenges of the use of exosomes as drug carrier for cancer therapy. Cancer Cell Int..

[23] Pan B.-T., Johnstone R.M. (1983). Fate of the transferrin receptor during maturation of sheep reticulocytes in vitro: Selective externalization of the receptor. Cell.

[24] Liu S., Wu X., Chandra S., Lyon C., Ning B., Jiang L., Fan J., Hu T.Y. (2022). Extracellular vesicles: Emerging tools as therapeutic agent carriers. Acta Pharm. Sin. B.

[25] Kalluri R., LeBleu V.S. (2020). The biology, function, and biomedical applications of exosomes. Science.

[26] Raposo G., Nijman H.W., Stoorvogel W., Liejendekker R., Harding C.V., Melief C.J., Geuze H.J. (1996). B lymphocytes secrete antigen-presenting vesicles. J. Exp. Med..

[27] Xu Z., Zeng S., Gong Z., Yan Y. (2020). Exosome-based immunotherapy: A promising approach for cancer treatment. Mol. Cancer.

[28] Zhang L., Yu D. (2019). Exosomes in cancer development, metastasis, and immunity. Biochim. Biophys. Acta (BBA) Rev. Cancer.

[29] Nam G., Choi Y., Kim G.B., Kim S., Kim A.S., Kim I. (2020). Emerging Prospects of Exosomes for Cancer Treatment: From Conventional Therapy to Immunotherapy. Adv. Mater..

[30] Jella K.K., Nasti T.H., Li Z., Malla S.R., Buchwald Z.S., Khan M.K. (2018). Exosomes, Their Biogenesis and Role in Inter-Cellular Communication, Tumor Microenvironment and Cancer Immunotherapy. Vaccines.

[31] Hanayama R. (2020). Emerging roles of extracellular vesicles in physiology and disease. J. Biochem..

[32] Zhang Y., Liu Y., Liu H., Tang W.H. (2019). Exosomes: Biogenesis, biologic function and clinical potential. Cell Biosci..

[33] Rezaie J., Ajezi S., Avci B., Karimipour M., Geranmayeh M.H., Nourazarian A., Sokullu E., Rezabakhsh A., Rahbarghazi R. (2017). Exosomes and their Application in Biomedical Field: Difficulties and Advantages. Mol. Neurobiol..

[34] Meldolesi J. (2018). Exosomes and Ectosomes in Intercellular Communication. Curr. Biol..

[35] Van Niel G., D’Angelo G., Raposo G. (2018). Shedding light on the cell biology of extracellular vesicles. Nat. Rev. Mol. Cell Biol..

[36] Zhu L., Sun H.-T., Wang S., Huang S.-L., Zheng Y., Wang C.-Q., Hu B.-Y., Qin W., Zou T.-T., Fu Y. (2020). Isolation and characterization of exosomes for cancer research. J. Hematol. Oncol..

[37] Zhao Y., Liu L., Sun R., Cui G., Guo S., Han S., Li Z., Bai T., Teng L. (2022). Exosomes in cancer immunoediting and immunotherapy. Asian J. Pharm. Sci..

[38] Thakur A., Ke X., Chen Y.-W., Motallebnejad P., Zhang K., Lian Q., Chen H.J. (2021). The mini player with diverse functions: Extracellular vesicles in cell biology, disease, and therapeutics. Protein Cell.

[39] Han Q.-F., Li W.-J., Hu K.-S., Gao J., Zhai W.-L., Yang J.-H., Zhang S.-J. (2022). Exosome biogenesis: Machinery, regulation, and therapeutic implications in cancer. Mol. Cancer.

[40] Leone A.D., Rees A.J., Kain R. (2018). Dendritic cells and routing cargo into exosomes. Immunol. Cell Biol..

[41] Chen X., Chi H., Zhao X., Pan R., Wei Y., Han Y. (2022). Role of Exosomes in Immune Microenvironment of Hepatocellular Carcinoma. J. Oncol..

[42] Qian K., Fu W., Li T., Zhao J., Lei C., Hu S. (2022). The roles of small extracellular vesicles in cancer and immune regulation and translational potential in cancer therapy. J. Exp. Clin. Cancer Res..

[43] Jennrich S., Pelzer M., Tertel T., Koska B., Vüllings M., Thakur B.K., Jendrossek V., Timmermann B., Giebel B., Rudner J. (2022). CD9- and CD81-positive extracellular vesicles provide a marker to monitor glioblastoma cell response to photon-based and proton-based radiotherapy. Front. Oncol..

[44] Wei D., Zhan W., Gao Y., Huang L., Gong R., Wang W., Zhang R., Wu Y., Gao S., Kang T. (2021). RAB31 marks and controls an ESCRT-independent exosome pathway. Cell Res..

[45] Deb A., Gupta S., Mazumder P.B. (2020). Exosomes: A new horizon in modern medicine. Life Sci..

[46] Yang E., Wang X., Gong Z., Yu M., Wu H., Zhang D. (2020). Exosome-mediated metabolic reprogramming: The emerging role in tumor microenvironment remodeling and its influence on cancer progression. Signal Transduct. Target. Ther..

[47] Jena B.C., Mandal M. (2020). The emerging roles of exosomes in anti-cancer drug resistance and tumor progression: An insight towards tumor-microenvironment interaction. Biochim. Biophys. Acta-Rev. Cancer.

[48] Théry C., Boussac M., Véron P., Ricciardi-Castagnoli P., Raposo G., Garin J., Amigorena S. (2001). Proteomic Analysis of Dendritic Cell-Derived Exosomes: A Secreted Subcellular Compartment Distinct from Apoptotic Vesicles. J. Immunol..

[49] Ginini L., Billan S., Fridman E., Gil Z. (2022). Insight into Extracellular Vesicle-Cell Communication: From Cell Recognition to Intracellular Fate. Cells.

[50] Wahlund C.J.E., Güclüler G., Hiltbrunner S., Veerman R.E., Näslund T.I., Gabrielsson S. (2017). Exosomes from antigen-pulsed dendritic cells induce stronger antigen-specific immune responses than microvesicles in vivo. Sci. Rep..

[51] Gao W., Liu H., Yuan J., Wu C., Huang D., Ma Y., Zhu J., Ma L., Guo J., Shi H. (2016). Exosomes derived from mature dendritic cells increase endothelial inflammation and atherosclerosis via membrane TNF-α mediated NF-κB pathway. J. Cell. Mol. Med..

[52] Munich S., Sobo-Vujanovic A., Buchser W.J., Beer-Stolz D., Vujanovic N.L. (2012). Dendritic cell exosomes directly kill tumor cells and activate natural killer cells via TNF superfamily ligands. Oncoimmunology.

[53] Liu Q., Rojas-Canales D.M., DiVito S.J., Shufesky W.J., Stolz D.B., Erdos G., Sullivan M.L., Gibson G.A., Watkins S.C., Larregina A.T. (2016). Donor dendritic cell–derived exosomes promote allograft-targeting immune response. J. Clin. Investig..

[54] O’brien K., Breyne K., Ughetto S., Laurent L.C., Breakefield X.O. (2020). RNA delivery by extracellular vesicles in mammalian cells and its applications. Nat. Rev. Mol. Cell Biol..

[55] Montecalvo A., Larregina A.T., Shufesky W.J., Beer Stolz D., Sullivan M.L.G., Karlsson J.M., Baty C.J., Gibson G.A., Erdos G., Wang Z. (2012). Mechanism of transfer of functional microRNAs between mouse dendritic cells via exosomes. Blood.

[56] Rezaie J., Feghhi M., Etemadi T. (2022). A review on exosomes application in clinical trials: Perspective, questions, and challenges. Cell Commun. Signal..

[57] Gurunathan S., Kang M.-H., Song H., Kim N.H., Kim J.-H. (2022). The role of extracellular vesicles in animal reproduction and diseases. J. Anim. Sci. Biotechnol..

[58] Maacha S., Bhat A.A., Jimenez L., Raza A., Haris M., Uddin S., Grivel J.-C. (2019). Extracellular vesicles-mediated intercellular communication: Roles in the tumor microenvironment and anti-cancer drug resistance. Mol. Cancer.

[59] Jugniot N., Dahl J.J., Paulmurugan R. (2022). Immunotheranostic microbubbles (iMBs)—A modular platform for dendritic cell vaccine delivery applied to breast cancer immunotherapy. J. Exp. Clin. Cancer Res..

[60] Viaud S., Théry C., Ploix S., Tursz T., Lapierre V., Lantz O., Zitvogel L., Chaput N. (2010). Dendritic Cell-Derived Exosomes for Cancer Immunotherapy: What’s Next?. Cancer Res..

[61] Tkach M., Kowal J., Zucchetti A.E., Enserink L., Jouve M., Lankar D., Saitakis M., Martin-Jaular L., Théry C. (2017). Qualitative differences in T-cell activation by dendritic cell-derived extracellular vesicle subtypes. EMBO J..

[62] Fu C., Zhou L., Mi Q.-S., Jiang A. (2020). DC-Based Vaccines for Cancer Immunotherapy. Vaccines.

[63] Pang X.-L., Wang Z.-G., Liu L., Feng Y.-H., Wang J.-X., Xie H.-C., Yang X.-L., Li J.-F., Feng G.-W. (2019). Immature dendritic cells derived exosomes promotes immune tolerance by regulating T cell differentiation in renal transplantation. Aging.

[64] Segura E., Nicco C., Lombard B., Véron P., Raposo G., Batteux F., Amigorena S., Théry C. (2005). ICAM-1 on exosomes from mature dendritic cells is critical for efficient naive T-cell priming. Blood.

[65] Zhang H., Tang K., Zhang Y., Ma R., Ma J., Li Y., Luo S., Liang X., Ji T., Gu Z. (2015). Cell-free Tumor Microparticle Vaccines Stimulate Dendritic Cells via cGAS/STING Signaling. Cancer Immunol. Res..

[66] Segura E., Amigorena S., Théry C. (2005). Mature dendritic cells secrete exosomes with strong ability to induce antigen-specific effector immune responses. Blood Cells Mol. Dis..

[67] Zhang E., Phan P., Zhao Z. (2023). Cellular nanovesicles for therapeutic immunomodulation: A perspective on engineering strategies and new advances. Acta Pharm. Sin. B.

[68] Chen Y.S., Lin E.Y., Chiou T.W., Harn H.J. (2020). Exosomes in clinical trial and their production in compliance with good manufacturing practice. Tzu-Chi Med. J..

[69] Mendt M., Kamerkar S., Sugimoto H., McAndrews K.M., Wu C.-C., Gagea M., Yang S., Blanko E.V.R., Peng Q., Ma X. (2018). Generation and testing of clinical-grade exosomes for pancreatic cancer. JCI Insight.

[70] Tel J., Aarntzen E.H., Baba T., Schreibelt G., Schulte B.M., Benitez-Ribas D., Boerman O.C., Croockewit S., Oyen W.J.G., van Rossum M. (2013). Natural human plasmacytoid dendritic cells induce antigen-specific T-cell responses in melanoma patients. Cancer Res..

[71] Charles J., Chaperot L., Hannani D., Costa J.B., Templier I., Trabelsi S., Gil H., Moisan A., Persoons V., Hegelhofer H. (2020). An innovative plasmacytoid dendritic cell line-based cancer vaccine primes and expands antitumor T-cells in melanoma patients in a first-in-human trial. Oncoimmunology.

[72] Romagnoli G.G., Zelante B.B., Toniolo P.A., Migliori I.K., Barbuto J.A.M. (2015). Dendritic Cell-Derived Exosomes may be a Tool for Cancer Immunotherapy by Converting Tumor Cells into Immunogenic Targets. Front. Immunol..

[73] Fu W., Li T., Chen H., Zhu S., Zhou C. (2022). Research Progress in Exosome-Based Nanoscale Drug Carriers in Tumor Therapies. Front. Oncol..

[74] Rehman F.U., Liu Y., Zheng M., Shi B. (2023). Exosomes based strategies for brain drug delivery. Biomaterials.

[75] Laoui D., Keirsse J., Morias Y., Van Overmeire E., Geeraerts X., Elkrim Y., Kiss M., Bolli E., Lahmar Q., Sichien D. (2016). The tumour microenvironment harbours ontogenically distinct dendritic cell populations with opposing effects on tumour immunity. Nat. Commun..

[76] Gupta D., Liang X., Pavlova S., Wiklander O.P., Corso G., Zhao Y., Saher O., Bost J., Zickler A.M., Piffko A. (2020). Quantification of extracellular vesicles in vitro and in vivo using sensitive bioluminescence imaging. J. Extracell. Vesicles.

[77] Shrestha B., Zhang Y., Yu B., Li G., Boucher J.C., Beatty N.J., Tsai H.-C., Wang X., Mishra A., Sweet K. (2019). Generation of Antitumor T Cells for Adoptive Cell Therapy with Artificial Antigen Presenting Cells. J. Immunother..

[78] Simhadri V.R., Reiners K.S., Hansen H.P., Topolar D., Simhadri V.L., Nohroudi K., Kufer T.A., Engert A., von Strandmann E.P. (2008). Dendritic Cells Release HLA-B-Associated Transcript-3 Positive Exosomes to Regulate Natural Killer Function. PLoS ONE.

[79] Zitvogel L., Regnault A., Lozier A., Wolfers J., Flament C., Tenza D., Ricciardi-Castagnoli P., Raposo G., Amigorena S. (1998). Eradication of established murine tumors using a novel cell-free vaccine: Dendritic cell derived exosomes. Nat. Med..

[80] Salmond N., Williams K.C. (2021). Isolation and characterization of extracellular vesicles for clinical applications in cancer—Time for standardization?. Nanoscale Adv..

[81] Veglia F., Gabrilovich I.D. (2017). Dendritic cells in cancer: The role revisited. Curr. Opin. Immunol..

[82] Cruz F.M., Colbert J.D., Merino E., Kriegsman B.A., Rock K.L. (2017). The Biology and Underlying Mechanisms of Cross-Presentation of Exogenous Antigens on MHC-I Molecules. Annual Review of Immunology..

[83] Xia L., Oyang L., Lin J., Tan S., Han Y., Wu N., Yi P., Tang L., Pan Q., Rao S. (2021). The cancer metabolic reprogramming and immune response. Mol. Cancer.

[84] Jhunjhunwala S., Hammer C., Delamarre L. (2021). Antigen presentation in cancer: Insights into tumour immunogenicity and immune evasion. Nat. Rev. Cancer.

[85] Viaud S., Terme M., Flament C., Taieb J., André F., Novault S., Escudier B., Robert C., Caillat-Zucman S., Tursz T. (2009). Dendritic Cell-Derived Exosomes Promote Natural Killer Cell Activation and Proliferation: A Role for NKG2D Ligands and IL-15Rα. PLoS ONE.

[86] Song H., Chen X., Hao Y., Wang J., Xie Q., Wang X. (2022). Nanoengineering facilitating the target mission: Targeted extracellular vesicles delivery systems design. J. Nanobiotechnol..

[87] Wakim L.M., Bevan M.J. (2011). Cross-dressed dendritic cells drive memory CD8^+^ T-cell activation after viral infection. Nature.

[88] Viaud S., Ploix S., Lapierre V., Théry C., Commere P.-H., Tramalloni D., Gorrichon K., Virault-Rocroy P., Tursz T., Lantz O. (2011). Updated Technology to Produce Highly Immunogenic Dendritic Cell-derived Exosomes of Clinical Grade. J. Immunother..

[89] Damo M., Wilson D.S., Simeoni E., Hubbell J.A. (2015). TLR-3 stimulation improves anti-tumor immunity elicited by dendritic cell exosome-based vaccines in a murine model of melanoma. Sci. Rep..

[90] Sobo-Vujanovic A., Munich S., Vujanovic N.L. (2014). Dendritic-cell exosomes cross-present Toll-like receptor-ligands and activate bystander dendritic cells. Cell. Immunol..

[91] Sheehan C., D’Souza-Schorey C. (2019). Tumor-derived extracellular vesicles: Molecular parcels that enable regulation of the immune response in cancer. J. Cell Sci..

[92] Näslund T.I., Gehrmann U., Qazi K.R., Karlsson M.C.I., Gabrielsson S. (2013). Dendritic Cell–Derived Exosomes Need to Activate Both T and B Cells To Induce Antitumor Immunity. J. Immunol..

[93] Alahdal M., Elkord E. (2022). Promising use of immune cell-derived exosomes in the treatment of SARS-CoV-2 infections. Clin. Transl. Med..

[94] Quah B.J.C., O’neill H.C. (2007). Mycoplasma contaminants present in exosome preparations induce polyclonal B cell responses. J. Leukoc. Biol..

[95] Qazi K.R., Gehrmann U., Domange Jordö E., Karlsson M.C.I., Gabrielsson S. (2009). Antigen-loaded exosomes alone induce Th1-type memory through a B cell–dependent mechanism. Blood.

[96] Colino J., Snapper C.M. (2006). Exosomes from Bone Marrow Dendritic Cells Pulsed with Diphtheria Toxoid Preferentially Induce Type 1 Antigen-Specific IgG Responses in Naive Recipients in the Absence of Free Antigen. J. Immunol..

[97] Tian H., Li W. (2017). Dendritic cell-derived exosomes for cancer immunotherapy: Hope and challenges. Ann. Transl. Med..

[98] Cao W., Chen Y., Han W., Yuan J., Xie W., Liu K., Qiu Y., Wang X., Li X. (2021). Potentiality of α-fetoprotein (AFP) and soluble intercellular adhesion molecule-1 (sICAM-1) in prognosis prediction and immunotherapy response for patients with hepatocellular carcinoma. Bioengineered.

[99] Lu Z., Zuo B., Jing R., Gao X., Rao Q., Liu Z., Qi H., Guo H., Yin H. (2017). Dendritic cell-derived exosomes elicit tumor regression in autochthonous hepatocellular carcinoma mouse models. J. Hepatol..

[100] Rao Q., Zuo B., Lu Z., Gao X., You A., Wu C., Du Z., Yin H. (2016). Tumor-derived exosomes elicit tumor suppression in murine hepatocellular carcinoma models and humans in vitro. Hepatology.

[101] Yang X., Chen J., Wang N., Liu Z., Li Y. (2018). Clinical use of dendritic cell-derived exosomes for hepatocellular carcinoma immunotherapy: How far we are?. J. Hepatol..

[102] Zhou L., Shen M., Fan X., Liu Y., Yang L. (2022). Pathogenic and Potential Therapeutic Roles of Exosomes Derived from Immune Cells in Liver Diseases. Front. Immunol..

[103] Shi S., Rao Q., Zhang C., Zhang X., Qin Y., Niu Z. (2018). Dendritic Cells Pulsed with Exosomes in Combination with PD-1 Antibody Increase the Efficacy of Sorafenib in Hepatocellular Carcinoma Model. Transl. Oncol..

[104] Liu C., Liu X., Xiang X., Pang X., Chen S., Zhang Y., Ren E., Zhang L., Liu X., Lv P. (2022). A nanovaccine for antigen self-presentation and immunosuppression reversal as a personalized cancer immunotherapy strategy. Nat. Nanotechnol..

[105] Poggio M., Hu T., Pai C.-C., Chu B., Belair C.D., Chang A., Montabana E., Lang U.E., Fu Q., Fong L. (2019). Suppression of exosomal PD-L1 induces systemic anti-tumor immunity and memory. Cell.

[106] Zhong X., Zhou Y., Cao Y., Ding J., Wang P., Luo Y., Liu H., Zhu Z., Jing X. (2020). Enhanced antitumor efficacy through microwave ablation combined with a dendritic cell-derived exosome vaccine in hepatocellular carcinoma. Int. J. Hyperth..

[107] Mathieu M., Névo N., Jouve M., Valenzuela J.I., Maurin M., Verweij F.J., Palmulli R., Lankar D., Dingli F., Loew D. (2021). Specificities of exosome versus small ectosome secretion revealed by live intracellular tracking of CD63 and CD9. Nat. Commun..

[108] Ruan S., Erwin N., He M. (2022). Light-induced high-efficient cellular production of immune functional extracellular vesicles. J. Extracell. Vesicles.

[109] Eisendle K., Weinlich G., Ebner S., Forstner M., Reider D., Zelle-Rieser C., Tripp C.H., Fritsch P., Stoitzner P., Romani N. (2020). Combining chemotherapy and autologous peptide-pulsed dendritic cells provides survival benefit in stage IV melanoma patients. JDDG J. Dtsch. Dermatol. Ges..

